# Modeling homeostasis mechanisms that set the target cell size

**DOI:** 10.1038/s41598-020-70923-0

**Published:** 2020-08-18

**Authors:** Cesar A. Vargas-Garcia, Mikael Björklund, Abhyudai Singh

**Affiliations:** 1 Corporación Colombiana de Investigación Agropecuaria-Agrosavia, Mosquera, Colombia; 2 Fundación Universitaria Konrad Lorenz, Bogotá, Colombia; 3 Zhejiang University-University of Edinburgh (ZJU-UoE) Institute, 718 East Haizhou Rd., Haining, 314400 Zhejiang People’s Republic of China; 4 Second Affiliated Hospital, Zhejiang University School of Medicine, Hangzhou, Zhejiang People’s Republic of China; 5 Department of Biomedical Engineering, University of Delaware, Newark, Delaware USA; 6 Department of Electrical and Computer Engineering, University of Delaware, Newark, Delaware USA; 7 Department of Mathematical Sciences, University of Delaware, Newark, Delaware USA

**Keywords:** Computational biophysics, Cell growth, Mitosis, Cell growth, Computational models, Applied mathematics

## Abstract

How organisms maintain cell size homeostasis is a long-standing problem that remains unresolved, especially in multicellular organisms. Recent experiments in diverse animal cell types demonstrate that within a cell population, cellular proliferation is low for small and large cells, but high at intermediate sizes. Here we use mathematical models to explore size-control strategies that drive such a non-monotonic profile resulting in the proliferation capacity being maximized at a target cell size. Our analysis reveals that most models of size control yield proliferation capacities that vary monotonically with cell size, and non-monotonicity requires two key mechanisms: (1) the growth rate decreases with increasing size for excessively large cells; and (2) cell division occurs as per the Adder model (division is triggered upon adding a fixed size from birth), or a Sizer-Adder combination. Consistent with theory, Jurkat T cell growth rates increase with size for small cells, but decrease with size for large cells. In summary, our models show that regulation of both growth and cell-division timing is necessary for size control in animal cells, and this joint mechanism leads to a target cell size where cellular proliferation capacity is maximized.

## Introduction

Cell size control is a fundamental aspect of biology observed at different domains of life, but in most cases remains poorly understood^3–9^. Two popular models for cell size control have been extensively studied and debated^10^. In the “sizer” or the size-checkpoint model, cell-cycle transitions occur after attainment of a minimum cell mass or size, whereas in the “adder” model cells add a fixed amount of size in each division cycle independent of the daughter size. Strong evidence for adder has been reported in a diverse set of prokaryotes^11–21^ and budding yeast^22^. In cultured mammalian cell lines both adder, sizer and intermediate models can be observed^23,24^, while in the mouse epidermis in vivo, a sizer behavior is apparent^25^.

**Figure 1:**
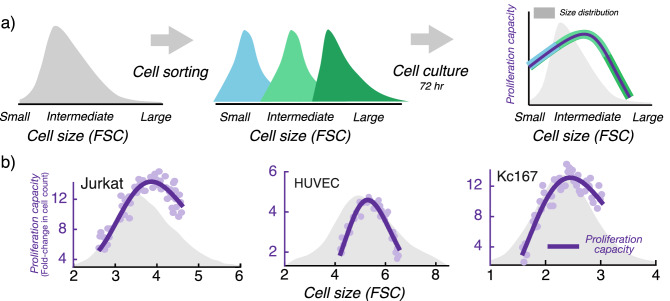
Cellular proliferation capacity is maximized at a target cell size. (**a**) Using forward scatter intensity (FSC) as a proxy for cell size^1,2^, flow cytometry is used to sort an original unsynchronized cell population (grey) into several subpopulations with different cell sizes. Each subpopulation is cultured for 72 h (approximately 3–5 cell generations), and proliferation capacity is quantified by measuring the relative change in cell counts. Interested readers are referred to the material and methods of^1^ for further details. (**b**) Measured proliferation capacity is plotted as a function of the average subpopulation FSC at the time of sorting for three different cell types: Jurkat cells (human T lymphocyte cell line), HUVEC (human umbilical vein endothelial cells; a primary cell line) and Kc167 (a widely used Drosophila cell line). The original cell size distribution is shown in grey. Experimental data presented here was taken from Fig. 3D of Miettinen and Björklund, Developmental Cell, 2016^1^.

Recent experiments in animal cells revealed an intriguing observation about cellular proliferation that ties deeply into size control, suggesting an evolutionary reason why cells aim to maintain a certain size. In these experiments^1^, cells within a population were first sorted into several subpopulations based on cell size, and then the net proliferation (relative fold-change in cell count) was quantified for each cultured subpopulation after 72 h. Throughout the paper we refer to this relative increase in cell counts as the *proliferation capacity*. Interestingly, data across several cell types shows a non-monotonic bell-shaped profile, where the proliferation capacity is maximized at a target cell size (Fig. 1). Importantly, apoptosis rates were similar for large and average-sized cells, and hence the decrease in proliferation at higher sizes is not simply due to elevated cell death^1,26–30^. Cellular proliferation measured via dye-dilution experiments showed identical trends for varying cells sizes (see supplementary figure S4 in^1^), providing another experimental confirmation for the existence of a target cell size where the proliferative capacity is maximal. A key focus of this work is to uncover cell size homeostasis mechanisms that drive such a non-monotonic proliferation profile. In particular, we explore what forms of size-based regulation of the cell growth rate, and the timing of mitosis, are necessary and sufficient for the proliferation capacity to be maximized at a target cell size. We proceed by first considering a deterministic formulation of the problem, followed by a systematic analysis ruling out incompatible size-control mechanisms, and finally identifying mechanisms consistent with experimental data.

## Problem formulation

In the experimental work of Miettinen and Björklund, Developmental Cell, 2016^1^, flow cytometry is used to sort an original cell population into several subpopulations with different cell sizes. Each subpopulation bin has a couple of thousand cells initially, and the relative fold-change in cell count is measured after 72 h. Given the very large number of cells present within each size bin, we take a deterministic approach that predicts that average number of divisions incurred by an individual cell within each cell size category.

In its most simplistic form, cell size control can be deterministically modeled as

1 $$\begin{aligned} V_{n+1}=\frac{aV_{n}+(2-a){\bar{V}}}{2}, \end{aligned}$$

where $V_n$ is the size of a newborn cell in the *n*th generation^22,31–33^, ${\bar{V}} > 0$ represents the discrete-time model’s unique fixed point, and parameter *a* takes values in [0, 2]. Here $aV_{n}+(2-a){\bar{V}}$ is the mother cell size just prior to mitosis, and symmetric division of a mother cell into two daughters yields the newborn size in the next generation. Specific values of *a* correspond to well-known strategies for regulating the timing of cell division:

2 $$\begin{aligned} a={\left\{ \begin{array}{ll} 0 &{}\quad \text {Sizer: cell division occurs at a fixed size threshold }2{\bar{V}}.\\ 1 &{}\quad \text {Adder: division occurs upon adding a fixed size }{\bar{V}}\text { from birth.}\\ 2 &{}\quad \text {Timer: for an exponentially growing cell, division occurs after a fixed time from birth.} \end{array}\right. } \end{aligned}$$

Values of *a* between zero and one imply an adder-sizer mixture, and such combinatorial control of cell size have been proposed in many organisms^24,32,34–36^. As per (1), given an initial newborn cell size $V_0$, the newborn sizes over generations evolve as

3 $$\begin{aligned} V_{n}={\bar{V}}+\left( \frac{a}{2}\right) ^{n}(V_{0}-{\bar{V}}), \quad n\in \{0,1,2,\ldots \}, \end{aligned}$$

and converge to $\lim _{n\rightarrow \infty } V_n={\bar{V}}$ for $0\le a < 2$ with convergence being faster for smaller values of *a*. For example, for a sizer ($a=0$) the correction in cell size happens in a single generation with $V_{n}={\bar{V}}, \ \ n\in \{1,2,\ldots \}$. Note that for $a=2$ there is no convergence to ${\bar{V}}$, i.e., $V_{n}=V_0, \ \ n\in \{0,1,2,\ldots \}$, and this corresponds to a neutrally-stable fixed point. Not surprisingly, $a=2$ is non-homeostatic in the sense that, the variance in the newborn size grows unboundedly over time in the presence of arbitrary small noise^37,38^.

The growth in cell size within a generation is described by the following ordinary differential equation

4 $$\begin{aligned} \frac{dv}{dt}=f(v),\quad v(0)=V_n \end{aligned}$$

where *v*(*t*) is the size of an individual cell at time *t* since the start of cell cycle and the function *f* describes a general size-dependent growth rate. We use this model to predict the cell-cycle durations across generations. Following the nomenclature used in^39^, we define the change in cell size per unit time as the growth rate. As per this definition, a constant *f* would correspond to a linear growth in cell size, and $f(v) \propto v$ would correspond to an exponential growth in cell size. Given $v(0)=V_n$ at the start of the cell cycle, the time taken to reach the mother cell size needed for division is

5 $$\begin{aligned} T(V_n)=\int _{V_{n}}^{aV_{n}+(2-a){\bar{V}}}\frac{dv}{f(v)}. \end{aligned}$$

We further generalize (5) to

6 $$\begin{aligned} T(V_n)=\max \left( T_{\text {min}},\int _{V_{n}}^{aV_{n}+(2-a){\bar{V}}}\frac{dv}{f(v)} \right) , \end{aligned}$$

constraining newborns to stay in the cell cycle for a minimal duration $T_{\text {min}}$ before mitosis can take place. In essence, our model captures two key features of size control: size-based regulation of cell growth (via function *f*) and size-based regulation of division timing (via parameter *a*). We next discuss how these features determine cellular proliferation as observed in Fig. 1.

Starting from a single newborn cell of size $V_0$, the number of cell cycles that occur in a fixed time duration $T_f$ is the maximum values of $N\in \{0,1,2,\ldots \}$ such that

7 $$\begin{aligned} \sum _{n=0}^{N} T(V_n) \le T_f, \end{aligned}$$

i.e., the maximum number of generations such that the sum of cell-cycle durations across generations is less than $T_f$. Given *a*, *f*, $T_{\text {min}}$, $T_f$, jointly solving (3), (6) and (7) quantifies the proliferation capacity $2^N$ for a given initial cell size $V_0$. For example, if $T(V_0)<T_f$ but $T(V_0)+T(V_1)>T_f$, then the cell will undergo only one round of doubling ($N=1$) to have two cells. Similarly, if $T(V_0)>T_f$ then $N=0$ and there will be no cell doublings. In the context of the data presented in Fig. 1, we are particularly interested in model features that lead to *N* varying non-monotonically with $V_0$. Our analysis shows that most models for size homeostasis cannot capture this non-monotonic behavior, and we identify selected scenarios that are consistent with it. To get analytical insights into the shape of *N* vs. $V_0$ we make two simplifying assumptions: $T_{\text {min}}=0$ and *N* is approximately obtained by solving the equation

8 $$\begin{aligned} \sum _{n=0}^{N} T(V_n) = T_f. \end{aligned}$$

Note that *N* obtained from the exact inequality (7) is essentially the floor of *N* (or the greatest integer less than or equal to *N*) obtained from the approximate equality (8). In other words, if we obtain $N=4.3$ from the equality (8), then the actual number of divisions based on the inequality (7) will be $N=4$. As the floor function is a monotonic function, the shape of *N* vs. $V_0$ is preserved between (7) and (8). Insights obtained from analytical results are illustrated by numerically computing *N* via (7) for a given $T_{\text {min}}>0$.

## Results

### Sizer-based cell division is incompatible with a non-monotonic proliferation profile

The sizer, where mitosis is triggered upon reaching a prescribed size threshold $2{\bar{V}}$, is perhaps the simplest (and the oldest proposed) mechanism for size homeostasis^40–42^. In this case, the duration of the first cell cycle

9 $$\begin{aligned} T(V_0)=\max \left( T_{\text {min}},\int _{V_{0}}^{2{\bar{V}}}\frac{dv}{f(v)} \right) , \end{aligned}$$

decreases with increasing initial size $V_0$. Recall that for a sizer, the newborn sizes and cell-cycle durations for all subsequent generations ($n \ge 1$) are constant and given by

10 $$\begin{aligned} T(V_n)=T({\bar{V}})=\max \left( T_{\text {min}},\int _{{\bar{V}}}^{2{\bar{V}}}\frac{dv}{f(v)} \right) , \quad n\in \{1,2,\ldots \}. \end{aligned}$$

Solving $\sum _{n=0}^{N} T(V_n) = T_f$ yields the following number of cell cycles incurred in a fixed time duration $T_f$

11 $$\begin{aligned} N=\frac{T_f-T(V_0)}{T({\bar{V}})}+1. \end{aligned}$$

As $T(V_0)$ is a decreasing function of $V_0$, the extent of proliferation $2^N$ increases with increasing $V_0$. Basically, a large cell quickly completes the first cell cycle, and has more time to complete subsequent rounds of replication. Thus, sizer-based division ($a=0$) is inconsistent with the non-monotonic cellular proliferation seen in Fig. 1, irrespective of how cellular growth is regulated via function *f*. Given the constraints on the parameter *a*, we next explore if similar constraints arise on the growth rate.

### Exponential or linear growth in cell size is incompatible with a non-monotonic proliferation profile

**Figure 2:**
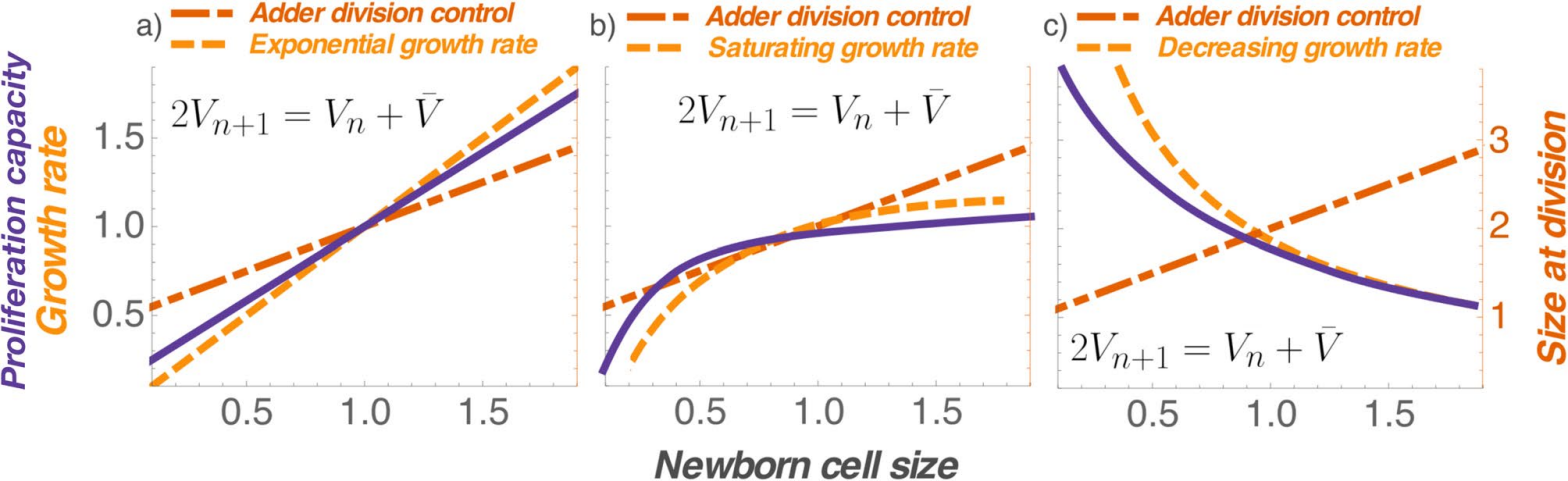
Monotonic growth rates coupled with Adder-based division control yield monotonic proliferation capacity profiles. Exponential growth (**a**), or an increasing growth rate followed by saturation (**b**) together with the Adder model always lead to a proliferation capacity monotonically increasing with size. In contrast, a decreasing growth rate together with an Adder yields a decreasing proliferation capacity (**c**). Newborn cell size (x axis) is normalized by its mean. Proliferation capacity, growth rate, and size at division (y axes) are normalized by their corresponding values for the mean newborn size. The proliferation capacity for the exponential growth case is computed by solving the equations (21)–(23) from the “Methods” section. In the saturating growth rate case, proliferation capacity is calculated using (24), (22), and (23) from the “Methods” section.

Let’s consider exponential growth in cell size during the cell cycle

12 $$\begin{aligned} \frac{dv}{dt}=\alpha v,\quad v(0)=V_n \end{aligned}$$

where $\alpha$ is the exponential growth coefficient. It follows from (3) and (5) that in this case

13 $$\begin{aligned} \sum _{n=0}^{N} T(V_n)=\frac{1}{\alpha }\log \left( a^N+\frac{\left( 2^{N}-a^{N}\right) {\bar{V}}}{V_{0}}\right) \end{aligned}$$

and the sum of cell-cycle durations is a decreasing function of $V_0$, but an increasing function of *N*. As a consequence, a larger sized initial cell will have to undergo many more cell cycles such that the sum $\sum _{n=0}^{N} T(V_n)$ remains fixed and equal to $T_f$. Thus, cellular proliferation increases with initial cell size irrespective of how division timing is regulated (i.e., the value of *a*). This point is exemplified in Fig. 2a for an adder ($a=1$) by plotting the proliferation capacity as a function of $V_0$ by numerically solving (7). An important implication of this result is that while exponential growth coupled with some form of division control may explain size homeostasis in prokaryotes and microbial eukaryotes, it does not yield the bell-shaped proliferation profiles seen in animal cells (Fig. 1).

Next we consider linear growth in cell size

14 $$\begin{aligned} \frac{dv}{dt}=\alpha ,\quad v(0)=V_n \end{aligned}$$

that yields

15 $$\begin{aligned} \sum _{n=0}^{N} T(V_n)=\frac{1}{\alpha }\sum _{n=0}^{N} (a-1)V_{n}+(2-a){\bar{V}}=\frac{N {\bar{V}}}{\alpha }+\frac{(a-1)(V_{0}-{\bar{V}})}{\alpha } \frac{1-\left( \frac{a}{2}\right) ^N}{1-\frac{a}{2}}. \end{aligned}$$

Interestingly, depending on the value of *a* one can get different monotonic profiles for the proliferation capacity. For a sizer ($a=0$), *N* always increases with increasing $V_{0}$. For an adder ($a=1$), the sum becomes invariant of $V_0$ leading to a constant $N={\alpha } T_f/ {\bar{V}}$. Finally, when $a>1$, the sum $\sum _{n=0}^{N} T(V_n)$ is an increasing function of both $V_0$ and *N*, and in order to keep it constant, *N* must decrease with increasing $V_0$. In summary, our analysis shows that both linear and exponential models of cell size dynamics cannot explain the non-monotonic proliferation capacity.

### Non-monotonic cell growth rate is necessary and sufficient to drive a non-monotonic proliferation capacity for an adder

We next focus our attention on the physiologically relevant case of the adder, where for $a=1$

16 $$\begin{aligned} V_{n+1}=\frac{V_{n}+{\bar{V}}}{2} \implies V_{n}={\bar{V}}+\frac{V_{0}-{\bar{V}}}{2^n}, \quad n\in \{0,1,2,\ldots \}. \end{aligned}$$

After a change in variables, the sum of the cell-cycle durations can be written as

17 $$\begin{aligned} \sum _{n=0}^{N} T(V_n)&= \sum _{n=0}^{N} \int _{V_{n}}^{V_{n}+{\bar{V}}}\frac{dv}{f(v)}\nonumber \\&=\sum _{n=0}^{N} \int _{0}^{{\bar{V}}}\frac{dz}{f(z+V_n)}\nonumber \\&=\int _{0}^{{\bar{V}}}\frac{dz}{f(z+V_0)}+\int _{0}^{{\bar{V}}} \frac{dz}{f\left( z+\frac{V_0+{\bar{V}}}{2}\right) }+\ldots +\int _{0}^{{\bar{V}}} \frac{dz}{f\left( z+{\bar{V}}+\frac{V_{0}-{\bar{V}}}{2^{N}},\right) }. \end{aligned}$$

**Figure 3:**
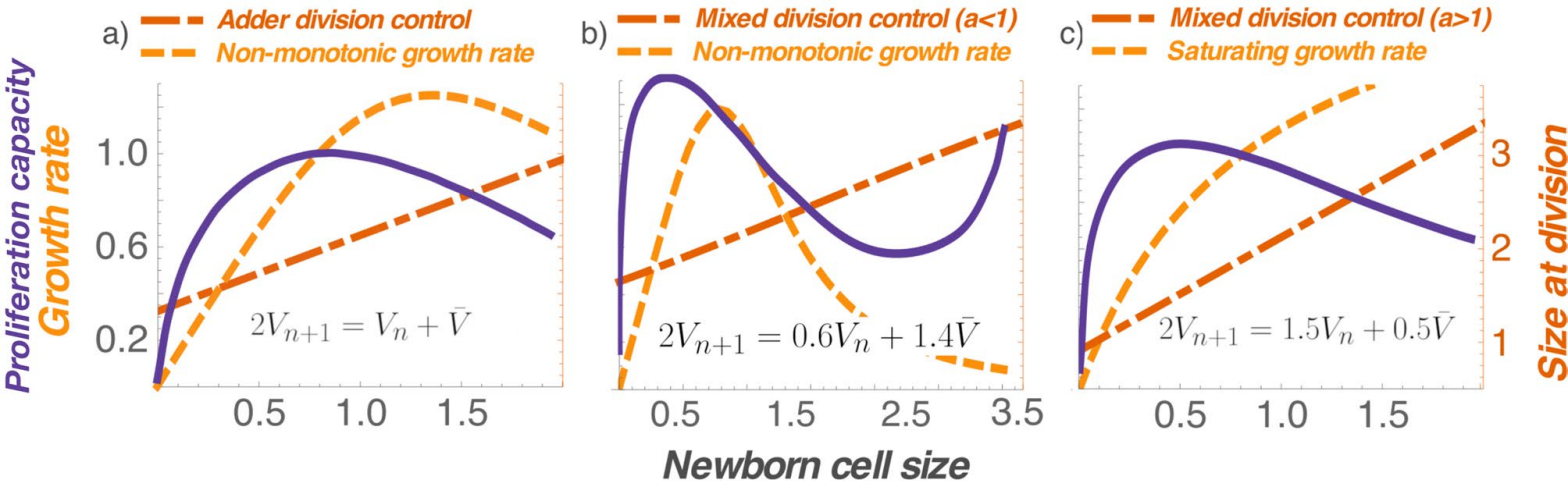
A non-monotonic growth rate coupled with Adder-based division control maximizes proliferation capacity at a target cell size. (**a**) A non-monotonic growth rate together with an Adder ($a=1$) yields a target cell size consistent with data in Fig. 1. (**b**) A non-monotonic growth rate together with an Adder-Sizer combination ($a<1$) results in a complex profile: a bell-shaped proliferation capacity for most physiological cell sizes, but it again increases at higher sizes. (**c**) A monotonically increasing growth rate can drive a bell-shaped proliferation capacity if $a>1$, i.e., size added in a cell-cycle duration increases with daughter cell size. Newborn cell size (x axis) is normalized by its mean. Proliferation capacity, growth rate, and size at division (y axes) are normalized by their corresponding values for the mean newborn size. The proliferation capacity for the saturating growth case is computed by solving the equations (24), (22), and (23) from the “Methods” section. For the non-monotonic growth rate case, proliferation capacity is calculated using (25), (22), and (23) from the “Methods” section.

As one can see, if *f* is a monotonically increasing function, then $\sum _{n=0}^{N} T(V_n)$ would be a decreasing function of $V_0$ (for fixed *N*), and an increasing function of *N* (for fixed $V_0$). Applying the same logic as before, *N* obtained by solving $\sum _{n=0}^{N} T(V_n) = T_f$ would an increasing function of $V_0$. Similarly, a monotonically decreasing function *f* will lead to *N* decreasing with increasing $V_0$. Thus, for an adder, monotonic growth rates yield monotonic proliferation capacity profiles (Fig. 2). Moreover, a non-monotonic proliferation capacity profile arises if the growth rate is non-monotonic. We illustrate this point with the following cell size dynamics

18 $$\begin{aligned} \frac{dv}{dt}=f(v)=\frac{\alpha v}{1+\left( {v}/{{V_{th}}}\right) ^k}, \end{aligned}$$

with two additional constants $k>1$ and ${V_{th}}>0$. Here growth is exponential for small newborns ($f \propto v$) and proliferation capacity increases with initial cell size. However, for large newborns, growth rate is decreasing ($f \propto v^{1-k}$), and proliferation capacity decreases with size (Fig. 3a). Interestingly, the non-monotonicity is preserved for values of *a* smaller than one, but with an additional feature—proliferation capacity can again increase for very large newborns (Fig. 3b). While such an increase is not seen in the data, it is possible that sizes needed for this effect fall outside the physiological range.

Finally, we point out that a non-monotonic proliferation capacity profile can arise for a monotonic growth rate, but this requires $a>1$, i.e., the size added from cell birth to division increases with newborn size. For example, consider a saturating growth rate

19 $$\begin{aligned} \frac{dv}{dt}=f(v)=\frac{\alpha v}{1+{v}/{{V_{th}}}}, \end{aligned}$$

that corresponds to $k=1$ in (18). As before, small newborns grow exponentially ($f(v)=\alpha v$) and proliferation capacity increases with size. Large newborns grow linearly ($f(v)=\alpha V_{th}$) and based on our earlier discussion on linear growth in cell size, proliferation capacity decreases with size when $a>1$ (Fig. 3c). Combining these two results, $a>1$ with a saturating growth rate results in a non-monotonic proliferation capacity profile.

## Discussion

Our results suggest that size-based regulation of both growth and timing of cell division are necessary determinants of a target cell size. This finding is consistent with experimental data from^23^, who showed that modulation of both growth rate and cell cycle duration is needed for size control in mammalian cells. However, joint regulation by itself is not sufficient to maximize cellular proliferation capacity, as our analysis of simple size control models reveals that monotonically increasing growth rate, coupled with any sizer-adder combination leads to proliferation capacity increasing with the initial cell size (Fig. 2). Importantly, our analysis rules out the following classical and popular explanations for cell size control as these are incompatible with a bell-shaped proliferation profile:

- Sizer-based regulation of cell-division timing, irrespective of the cellular growth rate *f*(*v*).
- Timer-based regulation of cell-division timing, irrespective of the cellular growth rate *f*(*v*). For a timer, the cell-cycle duration is invariant of the newborn size, and hence, the proliferation capacity will be independent of the initial cell size.
- Adder-based regulation of cell division timing with a cellular growth rate *f*(*v*) that varies monotonically with cell size.
- Exponential ($f \propto v$) or linear (*f* is constant) growth of cell size, irrespective of how division timing is regulated.

We uncover two scenarios that lead to cellular proliferation capacity being maximized at a target cell size. The first scenario involves a saturating growth rate of the form (19), where for small newborns the growth rate is proportional to size (i.e., exponential growth in cell size) and for large newborns the growth rate is a constant (i.e., linear growth in cell size). While such a growth rate together with a Timer (size-independent cell-cycle length) is sufficient for size homeostasis^37,38^, existence of a target cell size requires division timing to be controlled such that $a>1$ i.e., size added in each division cycle increases with newborn size (Fig. 3c). Note that increasing *a* beyond 1 reduces the stability of the fixed point $V_n={\bar{V}}$, that is ultimately destroyed at $a=2$. The reduced stability will manifest in larger newborn size fluctuations in the presence of noise^31^.

The second scenario involves a non-monotonic *f*(*v*) as in (18), where growth rate increases with cell size for small cells, but decreases with size for sufficiently large cell. Our analysis leads to a powerful result for adder-based control of mitosis timing—a non-monotonic growth rate is *necessary and sufficient* to drive a non-monotonic proliferation capacity profile (Fig. 3a), and these profiles persist for a sizer-adder mixture (Fig. 3b). Such decrease in growth rates for large cells has been reported in animal cells^28,29,39,43^, and is possibly attributed to a decreased surface area-to-volume ratio leading to insufficient nutrient exchange for supporting growth^44,45^. The growth rate of Jurkat T cells, as measured via oxygen consumption and mitochondrial activity, increase with size for small cells, but decrease with size for large cells^1^. Another recent study also showed that growing budding yeast and primary mammalian cells beyond a certain size leads to impaired cell-cycle progression, and the authors attribute this to cytoplasmic dilution or a reduced DNA to cytoplasm ratio, although in these cases the cells were grown substantially beyond their normal range^27^. Overall, the findings reported here are consistent with many experimental observations, including the recent single-cell tracking of several mammalian cell types, where the authors showed that size added in a cell-cycle duration is independent of the newborn size as per the adder model^23,46^.

In summary, our results uncover novel insights into size control principles, and provide a mechanistic explanation for the existence of a target size in proliferating animal cells. It is important to point that these findings are from cultured proliferating animal cells (spanning drosophila and human cells) as traditionally studied in the context of cell size control and there is no evidence that yeast and bacteria would behave similarly. These results will motivate other studies to validate and extend predictions beyond mammalian cells. For example, the unicellular alga *Chlamydomonas reinhardtii* has a very long G1 period, which allows cells to grow in size up to thirty fold^47^. In addition, single-cell expression profiling of large/small cells within the same population combined with mathematical modeling may also shed light on the molecular origins of non-monotonic growth rates^48–53^.

## Methods

To build Fig. 2a we assume the cell grows as per

20 $$\begin{aligned} \frac{dv}{dt}=\alpha v, \end{aligned}$$

where the growth rate was set to $\alpha \approx 1/48 \, h^{-1}$. Then we solve

21 $$\begin{aligned} T(V_n)=\max \left( T_{\text {min}}, \int _{V_n}^{2V_{n+1}}\frac{dv}{\alpha v}\right) , \end{aligned}$$

where the minimum cell cycle time was set to $T_{\text {min}}=20\, h$. Parameters *a* and ${\bar{V}}$ required to compute $V_n$ were set to 1 and 30 μm, respectively. We stop solving (14) until we meet the criteria for *N*

22 $$\begin{aligned} \sum _{n=0}^N T(V_n) \le T_f < \sum _{n=0}^{N+1} T(V_n), \end{aligned}$$

where the length of the experiment is $T_f=72 \, h$. We compute the proliferation capacity using

23 $$\begin{aligned} 2^{N+\frac{T_f-\sum _{n=0}^N T(V_n)}{T(V_{n+1})}}. \end{aligned}$$

where $\frac{T_f-\sum _{n=0}^N T(V_n)}{T(V_{n+1})}$ is a linear interpolator that connects *N* and $N+1$ divisions.

For the saturating growth rate case (Fig. 2b), we used

24 $$\begin{aligned} T(V_n)=\max \left( T_{\text {min}}, \int _{V_n}^{2V_{n+1}}\frac{1+{v}/{{V_{\text {th}}}}}{\alpha v}dv\right) , \end{aligned}$$

where $V_{\text {th}}=25$ μm. We performed the same steps as per the exponential growing case (2(a)) with same parameters. In Fig. 3c we use the same saturating growth rate but set $a=1.5$.

Figure 3a,b used the non-monotonic growth rate function and solve the equation

25 $$\begin{aligned} T(V_n)=\max \left( T_{\text {min}}, \int _{V_n}^{2V_{n+1}}\frac{1+({v}/{{V_{\text {th}}}})^k}{\alpha v}dv\right) , \end{aligned}$$

where $k=3$ and $V_{\text {th}}=20$ μm. We set $a=1$ in Fig. 3a and $a=0.6$ in Fig. 3b.
